# Bis[2-(isoquinolin-1-yl)phenyl-κ^2^*N*,*C*^1^](2-phenyl-1*H*-imidazo[4,5-*f*][1,10]phenanthroline-κ^2^*N*,*N*′)iridium(III) hexa­fluorido­phosphate methanol monosolvate

**DOI:** 10.1107/S2414314624008599

**Published:** 2024-09-06

**Authors:** Silas Martin, William W. Brennessel, Ammar Hasan, Carly R. Reed

**Affiliations:** a Department of Chemistry and Biochemistry State University of New York at Brockport Brockport NY 14420 USA; b Department of Chemistry 120 Trustee Road University of Rochester,Rochester NY 14627 USA; Benemérita Universidad Autónoma de Puebla, México

**Keywords:** crystal structure, iridium, 1-phenyl­iso­quinoline, 2-phenyl-1*H*-imidazo[4,5-*f*][1,10]phenanthroline, cyclo­metallated compound, organometallic compound

## Abstract

The title compound, [Ir(C_15_H_10_N)_2_(C_19_H_12_N_4_)]PF_6_·CH_3_OH, crystallizes in the *C*2/*c* space group with one monocationic iridium complex, one hexa­fluorido­phosphate anion, and one methanol solvent mol­ecule of crystallization in the asymmetric unit, all in general positions. The anion and solvent are linked to the iridium complex cation *via* hydrogen bonding. All bond lengths and angles fall into expected ranges compared to similar compounds.

## Abstract 2

The title iridium complex is the first X-ray characterized compound including both 1-phenyl­iso­quinoline and 2-phenyl-1*H*-imidazo[4,5-*f*][1,10]phenanthroline ligands.

## Graphical abstract

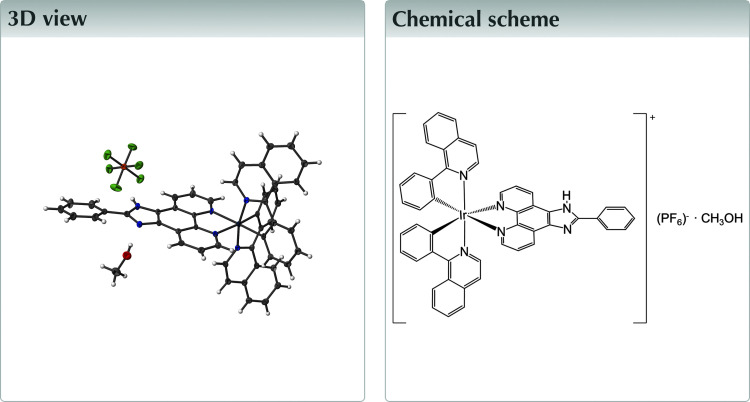

## Structure description

**Figure 1:**
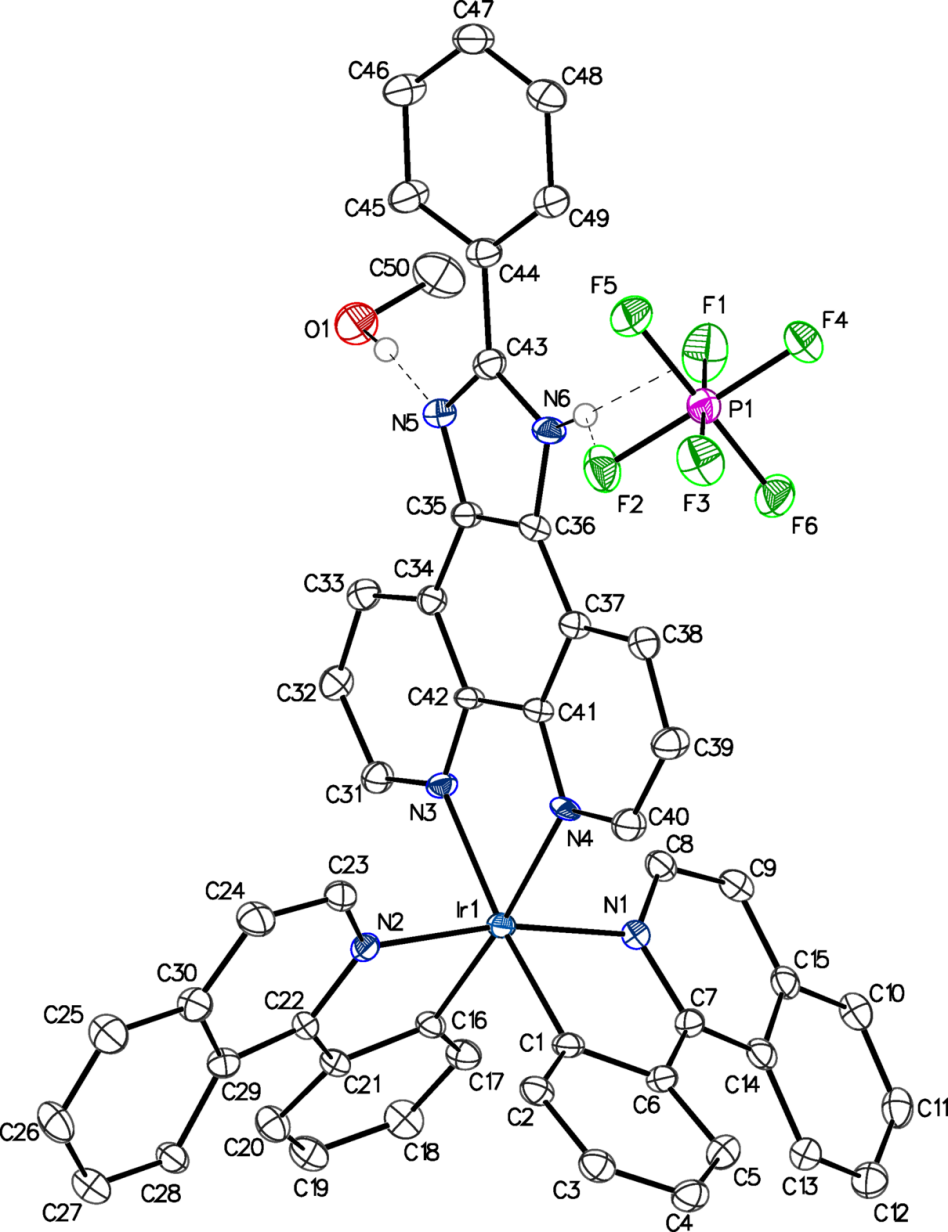
Anisotropic displacement ellipsoid plot of **1** drawn at the 50% probability level with C—H hydrogen atoms omitted. Dashed lines show hydrogen-bonding contacts, for which the N—H⋯F bond is bifurcated (see Table 2 ▸).

The solvent-free compound [Ir(C_15_H_10_N)_2_(C_19_H_12_N_4_)]PF_6_ has been previously synthesized, and its luminescent properties have been applied for carbon dioxide sensing (Ma *et al.*, 2015 ▸). Additionally, it has been found to act as an inhibitor of tumor necrosis factor-α (Kang *et al.*, 2016 ▸). In this study, we examined [Ir(C_15_H_10_N)_2_(C_19_H_12_N_4_)]PF_6_·CH_3_OH, **1**, which crystallizes in the *C*2/*c* space group. The asymmetric unit contains one monocationic iridium complex, one hexa­fluorido­phosphate anion, and one methanol solvent mol­ecule of crystallization, all in general positions (Fig. 1 ▸). The iridium atom is found at the center of the complex cation forming a distorted octa­hedral coordination environment (Table 1 ▸). A *cis*-*C*,*C* and *trans*-*N*,*N* configuration is observed in the chelating 1-phenyl­iso­quinoline ligands, as the nitro­gen atoms occupy the axial positions. The anion and solvent are linked to the iridium complex cation *via* bifurcated N—H⋯F and simple O—H⋯N hydrogen bonding, respectively (Fig. 1 ▸, Table 2 ▸). Similar bond lengths and angles to those of **1** are found in previously reported [Ir(1-phenyl­iso­quinoline)_2_(1,10-phenanthroline)](ClO_4_) (Zhao *et al.*, 2006 ▸) and [Ir(2-phenyl­pyridine)_2_(2-phenyl-1*H*-imidazo[4,5-*f*][1,10]phenanthroline)]PF_6_ (Zhao *et al.*, 2007 ▸).

## Synthesis and crystallization

Complex **1** was synthesized according to a previously published procedure (Ma *et al.*, 2015 ▸). Dark red crystals were obtained by dissolving **1** in a 5:1 di­chloro­methane:methanol solvent mixture and layering with diethyl ether.

## Refinement

Crystal data, data collection and structure refinement details are summarized in Table 3 ▸. The maximum and minimum residual peaks of 1.01 e Å^−3^ and of −1.32 e Å^−3^ are found 0.74 and 0.75 Å from atoms N4 and Ir1, respectively.

## Supplementary Material

Crystal structure: contains datablock(s) . DOI: 10.1107/S2414314624008599/bh4087sup1.cif

Structure factors: contains datablock(s) I. DOI: 10.1107/S2414314624008599/bh4087Isup2.hkl

CCDC reference: 2380883

Additional supporting information:  crystallographic information; 3D view; checkCIF report

## Figures and Tables

**Table 1:** Selected geometric parameters (Å, °)

Ir1—N1	2.054 (2)	Ir1—N4	2.155 (3)
Ir1—N2	2.048 (3)	Ir1—C1	2.006 (3)
Ir1—N3	2.146 (2)	Ir1—C16	2.018 (3)
			
N1—Ir1—N3	98.87 (10)	C1—Ir1—N3	173.03 (11)
N1—Ir1—N4	91.43 (10)	C1—Ir1—N4	96.27 (11)
N2—Ir1—N1	168.36 (10)	C1—Ir1—C16	88.89 (12)
N2—Ir1—N3	89.77 (9)	C16—Ir1—N1	91.39 (11)
N2—Ir1—N4	98.16 (10)	C16—Ir1—N2	79.62 (11)
N3—Ir1—N4	76.90 (10)	C16—Ir1—N3	97.98 (11)
C1—Ir1—N1	79.73 (11)	C16—Ir1—N4	174.49 (11)
C1—Ir1—N2	92.61 (11)		

**Table 2:** Hydrogen-bond geometry (Å, °)

*D*—H⋯*A*	*D*—H	H⋯*A*	*D*⋯*A*	*D*—H⋯*A*
N6—H6⋯F1	0.85 (5)	2.28 (5)	3.110 (4)	166 (4)
N6—H6⋯F2	0.85 (5)	2.57 (4)	3.249 (4)	139 (4)
O1—H1⋯N5	0.93 (7)	1.92 (7)	2.849 (4)	175 (6)

**Table 3:** Experimental details

Crystal data	
Chemical formula	[Ir(C_15_H_10_N)_2_(C_19_H_12_N_4_)]PF_6_·CH_4_O
*M*_r_	1074.02
Crystal system, space group	Monoclinic, *C*2/*c*
Temperature (K)	100
*a*, *b*, *c* (Å)	21.7603 (1), 19.6660 (1), 22.1646 (1)
β (°)	119.618 (1)
*V* (Å^3^)	8245.75 (10)
*Z*	8
Radiation type	Cu *K*α
μ (mm^−1^)	7.29
Crystal size (mm)	0.23 × 0.1 × 0.08

Data collection	
Diffractometer	XtaLAB Synergy, Dualflex, HyPix
Absorption correction	Multi-scan (*CrysAlis PRO*; Rigaku OD, 2023 ▸)
*T*_min_, *T*_max_	0.561, 1.000
No. of measured, independent and observed [*I* > 2σ(*I*)] reflections	259576, 8995, 8896
*R*_int_	0.048
(sin θ/λ)_max_ (Å^−1^)	0.640

Refinement	
*R*[*F*^2^ > 2σ(*F*^2^)], *wR*(*F*^2^), *S*	0.030, 0.078, 1.14
No. of reflections	8995
No. of parameters	595
H-atom treatment	H atoms treated by a mixture of independent and constrained refinement
Δρ_max_, Δρ_min_ (e Å^−3^)	1.01, −1.32

Computer programs: *CrysAlis PRO* (Rigaku OD, 2023 ▸), *SHELXT2018/2* (Sheldrick, 2015*a* ▸), *SHELXL2019/3* (Sheldrick, 2015*b* ▸) and *OLEX2* (Dolomanov *et al.*, 2009 ▸).

## full crystallographic data

### Bis[2-(isoquinolin-1-yl)phenyl-κ2N,C1](2-phenyl-1H-imidazo[4,5-f][1,10]phenanthroline-κ2N,N')iridium(III) hexafluoridophosphate methanol monosolvate Crystal data

**Table:**

[Ir(C_15_H_10_N)_2_(C_19_H_12_N_4_)]PF_6_·CH_4_O	*F*(000) = 4256
*M**_r_* = 1074.02	*D*_x_ = 1.730 Mg m^−^^3^
Monoclinic, *C*2/*c*	Cu *K*α radiation, λ = 1.54184 Å
*a* = 21.7603 (1) Å	Cell parameters from 182114 reflections
*b* = 19.6660 (1) Å	θ = 3.2–80.0°
*c* = 22.1646 (1) Å	µ = 7.29 mm^−^^1^
β = 119.618 (1)°	*T* = 100 K
*V* = 8245.75 (10) Å^3^	Block, red
*Z* = 8	0.23 × 0.1 × 0.08 mm

### Bis[2-(isoquinolin-1-yl)phenyl-κ2N,C1](2-phenyl-1H-imidazo[4,5-f][1,10]phenanthroline-κ2N,N')iridium(III) hexafluoridophosphate methanol monosolvate Data collection

**Table:**

XtaLAB Synergy, Dualflex, HyPix diffractometer	8896 reflections with *I* > 2σ(*I*)
Detector resolution: 10.0000 pixels mm^-1^	*R*_int_ = 0.048
ω scans	θ_max_ = 80.8°, θ_min_ = 3.2°
Absorption correction: multi-scan (CrysAlisPro; Rigaku OD, 2023)	*h* = −26→27
*T*_min_ = 0.561, *T*_max_ = 1.000	*k* = −25→25
259576 measured reflections	*l* = −28→28
8995 independent reflections	

### Bis[2-(isoquinolin-1-yl)phenyl-κ2N,C1](2-phenyl-1H-imidazo[4,5-f][1,10]phenanthroline-κ2N,N')iridium(III) hexafluoridophosphate methanol monosolvate Refinement

**Table:**

Refinement on *F*^2^	Primary atom site location: dual
Least-squares matrix: full	Secondary atom site location: difference Fourier map
*R*[*F*^2^ > 2σ(*F*^2^)] = 0.030	Hydrogen site location: mixed
*wR*(*F*^2^) = 0.078	H atoms treated by a mixture of independent and constrained refinement
*S* = 1.14	*w* = 1/[σ^2^(*F*_o_^2^) + (0.0335*P*)^2^ + 50.6715*P*] where *P* = (*F*_o_^2^ + 2*F*_c_^2^)/3
8995 reflections	(Δ/σ)_max_ = 0.002
595 parameters	Δρ_max_ = 1.01 e Å^−^^3^
0 restraints	Δρ_min_ = −1.32 e Å^−^^3^

### Bis[2-(isoquinolin-1-yl)phenyl-κ2N,C1](2-phenyl-1H-imidazo[4,5-f][1,10]phenanthroline-κ2N,N')iridium(III) hexafluoridophosphate methanol monosolvate Special details

**Table:**

Refinement. The O—H and N—H hydrogen atoms were found from a difference Fourier map and refined freely. All C—H atoms were placed geometrically and treated as riding atoms: aromatic/*sp*^2^, C—H = 0.95 Å with *U*_iso_(H) = 1.2*U*_eq_(C); methyl, C—H = 0.98 Å, with *U*_iso_(H) = 1.5*U*_eq_(C).

### Bis[2-(isoquinolin-1-yl)phenyl-κ2N,C1](2-phenyl-1H-imidazo[4,5-f][1,10]phenanthroline-κ2N,N')iridium(III) hexafluoridophosphate methanol monosolvate Fractional atomic coordinates and isotropic or equivalent isotropic displacement parameters (Å^2^)

**Table:**

	*x*	*y*	*z*	*U*_iso_*/*U*_eq_	
Ir1	0.18660 (2)	0.51478 (2)	0.22066 (2)	0.01310 (5)	
N1	0.18720 (13)	0.60590 (13)	0.26621 (13)	0.0145 (5)	
N2	0.16862 (13)	0.43208 (13)	0.15792 (13)	0.0149 (5)	
N3	0.20633 (13)	0.44817 (13)	0.30505 (12)	0.0144 (5)	
N4	0.30031 (13)	0.50921 (13)	0.27858 (13)	0.0147 (5)	
N5	0.40672 (14)	0.33569 (13)	0.50363 (13)	0.0172 (5)	
N6	0.48628 (14)	0.39005 (14)	0.48529 (13)	0.0173 (5)	
H6	0.526 (2)	0.401 (2)	0.489 (2)	0.026 (11)*	
C1	0.18075 (15)	0.57962 (16)	0.14815 (15)	0.0157 (6)	
C2	0.18909 (16)	0.56348 (16)	0.09124 (15)	0.0176 (6)	
H2	0.192633	0.517194	0.081070	0.021*	
C3	0.19226 (17)	0.61431 (17)	0.04933 (16)	0.0203 (6)	
H3	0.197584	0.602566	0.010587	0.024*	
C4	0.18768 (17)	0.68223 (17)	0.06385 (17)	0.0213 (6)	
H4	0.190386	0.716726	0.035248	0.026*	
C5	0.17921 (17)	0.69998 (17)	0.11978 (17)	0.0207 (6)	
H5	0.176968	0.746519	0.130022	0.025*	
C6	0.17395 (16)	0.64910 (15)	0.16112 (15)	0.0160 (6)	
C7	0.16784 (16)	0.66066 (15)	0.22381 (16)	0.0160 (6)	
C8	0.19469 (16)	0.60987 (16)	0.33094 (15)	0.0173 (6)	
H8	0.211764	0.571409	0.360673	0.021*	
C9	0.17829 (16)	0.66776 (17)	0.35421 (16)	0.0194 (6)	
H9	0.186885	0.670506	0.400536	0.023*	
C10	0.12331 (17)	0.78157 (17)	0.32736 (17)	0.0208 (6)	
H10	0.129581	0.784740	0.372835	0.025*	
C11	0.09020 (17)	0.83321 (17)	0.28119 (18)	0.0219 (6)	
H11	0.073887	0.872059	0.294600	0.026*	
C12	0.08044 (18)	0.82843 (17)	0.21359 (18)	0.0232 (7)	
H12	0.055001	0.863065	0.180716	0.028*	
C13	0.10717 (17)	0.77443 (16)	0.19446 (17)	0.0195 (6)	
H13	0.101974	0.773345	0.149323	0.023*	
C14	0.14248 (16)	0.72020 (16)	0.24142 (16)	0.0174 (6)	
C15	0.14841 (16)	0.72339 (16)	0.30850 (16)	0.0171 (6)	
C16	0.07999 (16)	0.51145 (15)	0.17072 (16)	0.0151 (6)	
C17	0.03519 (16)	0.54995 (16)	0.18522 (16)	0.0183 (6)	
H17	0.054671	0.583842	0.220293	0.022*	
C18	−0.03760 (17)	0.53975 (18)	0.14932 (17)	0.0220 (7)	
H18	−0.067336	0.567415	0.159181	0.026*	
C19	−0.06687 (18)	0.48936 (18)	0.09921 (18)	0.0225 (7)	
H19	−0.116544	0.482254	0.075198	0.027*	
C20	−0.02360 (17)	0.44914 (17)	0.08403 (17)	0.0203 (6)	
H20	−0.043410	0.413398	0.051067	0.024*	
C21	0.04973 (15)	0.46170 (16)	0.11777 (15)	0.0161 (6)	
C22	0.10093 (15)	0.42230 (15)	0.10704 (15)	0.0146 (5)	
C23	0.22179 (16)	0.39351 (16)	0.16016 (16)	0.0186 (6)	
H23	0.268933	0.401861	0.195821	0.022*	
C24	0.20981 (17)	0.34361 (18)	0.11330 (18)	0.0236 (7)	
H24	0.247386	0.314992	0.118743	0.028*	
C25	0.12723 (18)	0.28511 (18)	0.00484 (18)	0.0256 (7)	
H25	0.163464	0.254616	0.010214	0.031*	
C26	0.06167 (19)	0.28113 (19)	−0.05280 (18)	0.0261 (7)	
H26	0.051504	0.246443	−0.086268	0.031*	
C27	0.00949 (18)	0.32863 (18)	−0.06218 (17)	0.0232 (7)	
H27	−0.034475	0.328342	−0.104222	0.028*	
C28	0.02072 (16)	0.37537 (16)	−0.01193 (16)	0.0186 (6)	
H28	−0.015625	0.406575	−0.019150	0.022*	
C29	0.08624 (16)	0.37742 (16)	0.05072 (16)	0.0167 (6)	
C30	0.14120 (17)	0.33454 (17)	0.05647 (17)	0.0201 (6)	
C31	0.15769 (16)	0.41704 (16)	0.31510 (16)	0.0176 (6)	
H31	0.109209	0.426945	0.284676	0.021*	
C32	0.17553 (17)	0.37014 (16)	0.36901 (16)	0.0189 (6)	
H32	0.139528	0.348196	0.374208	0.023*	
C33	0.24523 (17)	0.35629 (16)	0.41403 (16)	0.0177 (6)	
H33	0.257901	0.325045	0.451028	0.021*	
C34	0.29790 (16)	0.38846 (15)	0.40528 (15)	0.0149 (6)	
C35	0.37268 (16)	0.37856 (15)	0.44779 (15)	0.0156 (6)	
C36	0.42059 (16)	0.41306 (16)	0.43528 (15)	0.0165 (6)	
C37	0.40060 (16)	0.46029 (16)	0.37990 (15)	0.0163 (6)	
C38	0.44647 (18)	0.49716 (17)	0.36460 (17)	0.0200 (6)	
H38	0.496217	0.494251	0.394022	0.024*	
C39	0.41821 (17)	0.53764 (18)	0.30623 (18)	0.0224 (7)	
H39	0.448525	0.562402	0.294778	0.027*	
C40	0.34468 (17)	0.54226 (17)	0.26377 (17)	0.0204 (6)	
H40	0.325949	0.569747	0.223272	0.024*	
C41	0.32684 (16)	0.46881 (15)	0.33610 (15)	0.0146 (5)	
C42	0.27599 (15)	0.43432 (15)	0.34924 (14)	0.0135 (5)	
C43	0.47508 (17)	0.34314 (16)	0.52491 (16)	0.0177 (6)	
C44	0.53159 (17)	0.30699 (16)	0.58436 (16)	0.0184 (6)	
C45	0.51344 (18)	0.25287 (18)	0.61353 (18)	0.0242 (7)	
H45	0.465587	0.238572	0.593134	0.029*	
C46	0.56512 (18)	0.22004 (18)	0.67214 (18)	0.0256 (7)	
H46	0.552253	0.184010	0.692242	0.031*	
C47	0.63527 (19)	0.23935 (18)	0.70155 (17)	0.0262 (7)	
H47	0.670585	0.216890	0.741742	0.031*	
C48	0.65336 (19)	0.29211 (19)	0.67145 (19)	0.0290 (8)	
H48	0.701460	0.305317	0.690984	0.035*	
C49	0.60218 (18)	0.32549 (18)	0.61349 (18)	0.0245 (7)	
H49	0.615347	0.361333	0.593424	0.029*	
P1	0.65912 (4)	0.44350 (4)	0.46518 (4)	0.01995 (16)	
F1	0.63651 (15)	0.44204 (13)	0.52467 (13)	0.0433 (6)	
F2	0.58550 (11)	0.40494 (11)	0.41450 (12)	0.0324 (5)	
F3	0.67841 (13)	0.44457 (13)	0.40529 (12)	0.0361 (5)	
F4	0.73098 (12)	0.48095 (11)	0.51717 (12)	0.0333 (5)	
F5	0.69646 (12)	0.37120 (11)	0.49073 (13)	0.0375 (5)	
F6	0.62004 (12)	0.51513 (10)	0.43986 (13)	0.0338 (5)	
O1	0.34705 (14)	0.28224 (14)	0.58277 (14)	0.0299 (5)	
H1	0.369 (3)	0.300 (3)	0.559 (3)	0.08 (2)*	
C50	0.3715 (2)	0.3233 (2)	0.6429 (2)	0.0382 (9)	
H50A	0.363207	0.371312	0.629379	0.057*	
H50B	0.422282	0.315711	0.673445	0.057*	
H50C	0.346082	0.311298	0.667589	0.057*	

### Bis[2-(isoquinolin-1-yl)phenyl-κ2N,C1](2-phenyl-1H-imidazo[4,5-f][1,10]phenanthroline-κ2N,N')iridium(III) hexafluoridophosphate methanol monosolvate Atomic displacement parameters (Å^2^)

**Table:**

	*U*^11^	*U*^22^	*U*^33^	*U*^12^	*U*^13^	*U*^23^
Ir1	0.01145 (7)	0.01431 (8)	0.01156 (7)	0.00028 (4)	0.00417 (5)	0.00151 (4)
N1	0.0140 (12)	0.0143 (12)	0.0141 (11)	−0.0007 (9)	0.0059 (10)	−0.0004 (9)
N2	0.0148 (12)	0.0123 (11)	0.0165 (12)	0.0009 (9)	0.0070 (10)	0.0017 (9)
N3	0.0158 (12)	0.0151 (12)	0.0118 (11)	0.0010 (9)	0.0065 (10)	0.0036 (9)
N4	0.0062 (11)	0.0182 (13)	0.0135 (12)	0.0002 (9)	0.0001 (9)	0.0019 (9)
N5	0.0163 (12)	0.0172 (12)	0.0161 (12)	0.0019 (10)	0.0064 (10)	0.0022 (10)
N6	0.0118 (12)	0.0197 (12)	0.0162 (12)	0.0012 (10)	0.0037 (10)	0.0036 (10)
C1	0.0122 (13)	0.0180 (14)	0.0135 (13)	0.0008 (11)	0.0037 (11)	0.0040 (11)
C2	0.0169 (14)	0.0218 (15)	0.0129 (13)	−0.0011 (11)	0.0064 (12)	0.0007 (11)
C3	0.0189 (15)	0.0253 (16)	0.0168 (14)	−0.0016 (12)	0.0088 (12)	0.0005 (12)
C4	0.0219 (16)	0.0237 (16)	0.0189 (15)	−0.0008 (13)	0.0105 (13)	0.0053 (12)
C5	0.0227 (16)	0.0179 (15)	0.0222 (15)	0.0001 (12)	0.0117 (13)	0.0024 (12)
C6	0.0154 (14)	0.0155 (14)	0.0146 (13)	0.0012 (11)	0.0056 (11)	0.0032 (11)
C7	0.0138 (13)	0.0143 (14)	0.0170 (14)	0.0002 (11)	0.0055 (11)	0.0017 (11)
C8	0.0168 (14)	0.0195 (15)	0.0137 (13)	−0.0024 (11)	0.0062 (12)	−0.0014 (11)
C9	0.0163 (14)	0.0247 (16)	0.0152 (14)	−0.0021 (12)	0.0061 (12)	−0.0012 (12)
C10	0.0177 (15)	0.0229 (16)	0.0210 (15)	−0.0026 (12)	0.0091 (13)	−0.0047 (12)
C11	0.0183 (15)	0.0186 (15)	0.0297 (17)	−0.0026 (12)	0.0126 (13)	−0.0041 (13)
C12	0.0197 (16)	0.0216 (16)	0.0267 (17)	0.0017 (12)	0.0102 (14)	0.0008 (13)
C13	0.0192 (15)	0.0171 (14)	0.0195 (15)	−0.0008 (12)	0.0075 (12)	0.0008 (12)
C14	0.0131 (13)	0.0203 (15)	0.0160 (14)	−0.0038 (11)	0.0049 (11)	−0.0013 (11)
C15	0.0135 (13)	0.0175 (14)	0.0181 (14)	−0.0033 (11)	0.0062 (11)	−0.0021 (11)
C16	0.0103 (13)	0.0174 (15)	0.0141 (14)	−0.0013 (10)	0.0033 (11)	0.0020 (11)
C17	0.0165 (14)	0.0208 (15)	0.0161 (14)	0.0029 (12)	0.0070 (12)	−0.0001 (12)
C18	0.0192 (15)	0.0276 (17)	0.0241 (16)	0.0007 (13)	0.0144 (13)	−0.0018 (13)
C19	0.0165 (15)	0.0296 (18)	0.0197 (16)	−0.0009 (13)	0.0076 (13)	−0.0011 (13)
C20	0.0162 (15)	0.0232 (16)	0.0212 (15)	−0.0008 (12)	0.0090 (13)	−0.0035 (12)
C21	0.0117 (13)	0.0175 (14)	0.0146 (13)	−0.0003 (11)	0.0032 (11)	0.0000 (11)
C22	0.0131 (13)	0.0134 (13)	0.0136 (13)	−0.0018 (11)	0.0039 (11)	−0.0004 (11)
C23	0.0140 (14)	0.0173 (14)	0.0198 (15)	0.0017 (11)	0.0047 (12)	0.0000 (12)
C24	0.0160 (15)	0.0248 (16)	0.0251 (16)	0.0042 (12)	0.0065 (13)	−0.0014 (13)
C25	0.0222 (17)	0.0268 (17)	0.0265 (17)	0.0042 (13)	0.0110 (14)	−0.0050 (14)
C26	0.0246 (17)	0.0294 (18)	0.0215 (16)	−0.0027 (14)	0.0091 (14)	−0.0106 (14)
C27	0.0183 (15)	0.0295 (17)	0.0188 (15)	−0.0011 (13)	0.0069 (13)	−0.0029 (13)
C28	0.0159 (14)	0.0204 (15)	0.0153 (14)	−0.0003 (11)	0.0047 (12)	−0.0021 (11)
C29	0.0168 (14)	0.0175 (14)	0.0160 (14)	0.0008 (11)	0.0084 (12)	0.0013 (11)
C30	0.0201 (15)	0.0201 (15)	0.0203 (15)	0.0026 (12)	0.0101 (13)	−0.0009 (12)
C31	0.0171 (14)	0.0179 (14)	0.0182 (14)	−0.0007 (11)	0.0089 (12)	0.0017 (12)
C32	0.0194 (15)	0.0191 (15)	0.0213 (15)	−0.0004 (12)	0.0124 (13)	0.0015 (12)
C33	0.0203 (15)	0.0168 (14)	0.0172 (14)	0.0029 (12)	0.0102 (12)	0.0008 (11)
C34	0.0151 (14)	0.0146 (13)	0.0147 (13)	0.0005 (11)	0.0072 (11)	−0.0001 (11)
C35	0.0157 (14)	0.0151 (14)	0.0127 (13)	0.0024 (11)	0.0045 (11)	0.0017 (11)
C36	0.0141 (14)	0.0185 (14)	0.0130 (13)	0.0002 (11)	0.0037 (11)	0.0002 (11)
C37	0.0143 (14)	0.0170 (14)	0.0143 (13)	0.0012 (11)	0.0046 (11)	0.0010 (11)
C38	0.0177 (15)	0.0196 (14)	0.0195 (15)	−0.0033 (12)	0.0068 (13)	0.0007 (12)
C39	0.0171 (15)	0.0249 (17)	0.0242 (16)	0.0000 (13)	0.0095 (13)	0.0081 (13)
C40	0.0173 (15)	0.0220 (16)	0.0203 (15)	−0.0022 (12)	0.0082 (13)	0.0041 (12)
C41	0.0124 (14)	0.0154 (13)	0.0117 (13)	0.0014 (11)	0.0027 (11)	0.0014 (11)
C42	0.0124 (13)	0.0142 (13)	0.0099 (12)	0.0028 (10)	0.0023 (11)	0.0022 (10)
C43	0.0202 (15)	0.0167 (14)	0.0162 (14)	0.0015 (12)	0.0091 (12)	0.0014 (11)
C44	0.0196 (15)	0.0189 (15)	0.0157 (14)	0.0050 (12)	0.0081 (12)	0.0016 (11)
C45	0.0215 (16)	0.0236 (16)	0.0263 (17)	0.0042 (13)	0.0108 (14)	0.0082 (13)
C46	0.0240 (17)	0.0277 (17)	0.0234 (16)	0.0049 (14)	0.0105 (14)	0.0084 (14)
C47	0.0273 (18)	0.0261 (17)	0.0180 (15)	0.0060 (14)	0.0056 (14)	0.0036 (13)
C48	0.0177 (16)	0.0293 (18)	0.0279 (18)	−0.0004 (14)	0.0020 (14)	0.0047 (15)
C49	0.0217 (16)	0.0208 (16)	0.0243 (16)	0.0002 (13)	0.0062 (14)	0.0047 (13)
P1	0.0199 (4)	0.0197 (4)	0.0203 (4)	−0.0003 (3)	0.0099 (3)	−0.0011 (3)
F1	0.0672 (17)	0.0390 (13)	0.0444 (14)	−0.0122 (12)	0.0433 (14)	−0.0067 (11)
F2	0.0216 (10)	0.0284 (11)	0.0422 (12)	−0.0034 (8)	0.0119 (9)	−0.0080 (9)
F3	0.0401 (13)	0.0451 (13)	0.0316 (11)	−0.0054 (10)	0.0243 (10)	−0.0069 (10)
F4	0.0262 (11)	0.0322 (12)	0.0299 (11)	−0.0052 (9)	0.0050 (9)	−0.0044 (9)
F5	0.0329 (12)	0.0246 (11)	0.0421 (13)	0.0048 (9)	0.0087 (10)	0.0037 (9)
F6	0.0283 (12)	0.0234 (11)	0.0429 (13)	0.0029 (8)	0.0123 (10)	0.0012 (9)
O1	0.0318 (14)	0.0317 (14)	0.0290 (13)	−0.0014 (11)	0.0171 (11)	0.0015 (11)
C50	0.037 (2)	0.051 (3)	0.0280 (19)	−0.0005 (19)	0.0174 (17)	−0.0025 (18)

### Bis[2-(isoquinolin-1-yl)phenyl-κ2N,C1](2-phenyl-1H-imidazo[4,5-f][1,10]phenanthroline-κ2N,N')iridium(III) hexafluoridophosphate methanol monosolvate Geometric parameters (Å, º)

**Table:**

Ir1—N1	2.054 (2)	C22—C29	1.430 (4)
Ir1—N2	2.048 (3)	C23—H23	0.9500
Ir1—N3	2.146 (2)	C23—C24	1.357 (5)
Ir1—N4	2.155 (3)	C24—H24	0.9500
Ir1—C1	2.006 (3)	C24—C30	1.410 (4)
Ir1—C16	2.018 (3)	C25—H25	0.9500
N1—C7	1.353 (4)	C25—C26	1.368 (5)
N1—C8	1.364 (4)	C25—C30	1.415 (5)
N2—C22	1.355 (4)	C26—H26	0.9500
N2—C23	1.364 (4)	C26—C27	1.404 (5)
N3—C31	1.333 (4)	C27—H27	0.9500
N3—C42	1.365 (4)	C27—C28	1.370 (5)
N4—C40	1.333 (4)	C28—H28	0.9500
N4—C41	1.364 (4)	C28—C29	1.416 (4)
N5—C35	1.373 (4)	C29—C30	1.417 (4)
N5—C43	1.328 (4)	C31—H31	0.9500
N6—H6	0.85 (5)	C31—C32	1.404 (4)
N6—C36	1.382 (4)	C32—H32	0.9500
N6—C43	1.376 (4)	C32—C33	1.369 (4)
C1—C2	1.397 (4)	C33—H33	0.9500
C1—C6	1.419 (4)	C33—C34	1.404 (4)
C2—H2	0.9500	C34—C35	1.434 (4)
C2—C3	1.389 (4)	C34—C42	1.414 (4)
C3—H3	0.9500	C35—C36	1.382 (4)
C3—C4	1.389 (5)	C36—C37	1.425 (4)
C4—H4	0.9500	C37—C38	1.404 (4)
C4—C5	1.385 (5)	C37—C41	1.416 (4)
C5—H5	0.9500	C38—H38	0.9500
C5—C6	1.399 (4)	C38—C39	1.378 (5)
C6—C7	1.477 (4)	C39—H39	0.9500
C7—C14	1.429 (4)	C39—C40	1.402 (4)
C8—H8	0.9500	C40—H40	0.9500
C8—C9	1.368 (4)	C41—C42	1.445 (4)
C9—H9	0.9500	C43—C44	1.467 (4)
C9—C15	1.412 (4)	C44—C45	1.400 (5)
C10—H10	0.9500	C44—C49	1.389 (5)
C10—C11	1.367 (5)	C45—H45	0.9500
C10—C15	1.417 (4)	C45—C46	1.387 (5)
C11—H11	0.9500	C46—H46	0.9500
C11—C12	1.409 (5)	C46—C47	1.384 (5)
C12—H12	0.9500	C47—H47	0.9500
C12—C13	1.375 (5)	C47—C48	1.392 (5)
C13—H13	0.9500	C48—H48	0.9500
C13—C14	1.422 (4)	C48—C49	1.381 (5)
C14—C15	1.429 (4)	C49—H49	0.9500
C16—C17	1.392 (4)	P1—F1	1.619 (2)
C16—C21	1.416 (4)	P1—F2	1.620 (2)
C17—H17	0.9500	P1—F3	1.578 (2)
C17—C18	1.392 (4)	P1—F4	1.592 (2)
C18—H18	0.9500	P1—F5	1.596 (2)
C18—C19	1.386 (5)	P1—F6	1.597 (2)
C19—H19	0.9500	O1—H1	0.93 (7)
C19—C20	1.392 (5)	O1—C50	1.419 (5)
C20—H20	0.9500	C50—H50A	0.9800
C20—C21	1.409 (4)	C50—H50B	0.9800
C21—C22	1.470 (4)	C50—H50C	0.9800
			
N1—Ir1—N3	98.87 (10)	C23—C24—H24	120.2
N1—Ir1—N4	91.43 (10)	C23—C24—C30	119.6 (3)
N2—Ir1—N1	168.36 (10)	C30—C24—H24	120.2
N2—Ir1—N3	89.77 (9)	C26—C25—H25	119.9
N2—Ir1—N4	98.16 (10)	C26—C25—C30	120.2 (3)
N3—Ir1—N4	76.90 (10)	C30—C25—H25	119.9
C1—Ir1—N1	79.73 (11)	C25—C26—H26	120.2
C1—Ir1—N2	92.61 (11)	C25—C26—C27	119.6 (3)
C1—Ir1—N3	173.03 (11)	C27—C26—H26	120.2
C1—Ir1—N4	96.27 (11)	C26—C27—H27	119.3
C1—Ir1—C16	88.89 (12)	C28—C27—C26	121.3 (3)
C16—Ir1—N1	91.39 (11)	C28—C27—H27	119.3
C16—Ir1—N2	79.62 (11)	C27—C28—H28	119.8
C16—Ir1—N3	97.98 (11)	C27—C28—C29	120.3 (3)
C16—Ir1—N4	174.49 (11)	C29—C28—H28	119.8
C7—N1—Ir1	115.4 (2)	C28—C29—C22	123.8 (3)
C7—N1—C8	121.3 (3)	C28—C29—C30	117.9 (3)
C8—N1—Ir1	122.4 (2)	C30—C29—C22	118.3 (3)
C22—N2—Ir1	116.2 (2)	C24—C30—C25	121.2 (3)
C22—N2—C23	120.3 (3)	C24—C30—C29	118.6 (3)
C23—N2—Ir1	122.9 (2)	C25—C30—C29	120.1 (3)
C31—N3—Ir1	126.3 (2)	N3—C31—H31	118.8
C31—N3—C42	118.7 (3)	N3—C31—C32	122.4 (3)
C42—N3—Ir1	114.84 (19)	C32—C31—H31	118.8
C40—N4—Ir1	125.9 (2)	C31—C32—H32	120.3
C40—N4—C41	119.4 (3)	C33—C32—C31	119.5 (3)
C41—N4—Ir1	114.7 (2)	C33—C32—H32	120.3
C43—N5—C35	105.2 (3)	C32—C33—H33	120.1
C36—N6—H6	126 (3)	C32—C33—C34	119.7 (3)
C43—N6—H6	127 (3)	C34—C33—H33	120.1
C43—N6—C36	107.0 (3)	C33—C34—C35	125.9 (3)
C2—C1—Ir1	126.4 (2)	C33—C34—C42	117.7 (3)
C2—C1—C6	118.3 (3)	C42—C34—C35	116.4 (3)
C6—C1—Ir1	114.9 (2)	N5—C35—C34	127.4 (3)
C1—C2—H2	119.6	N5—C35—C36	111.0 (3)
C3—C2—C1	120.8 (3)	C36—C35—C34	121.7 (3)
C3—C2—H2	119.6	N6—C36—C37	131.2 (3)
C2—C3—H3	119.9	C35—C36—N6	105.2 (3)
C4—C3—C2	120.3 (3)	C35—C36—C37	123.6 (3)
C4—C3—H3	119.9	C38—C37—C36	126.4 (3)
C3—C4—H4	119.8	C38—C37—C41	118.2 (3)
C5—C4—C3	120.4 (3)	C41—C37—C36	115.3 (3)
C5—C4—H4	119.8	C37—C38—H38	120.5
C4—C5—H5	120.1	C39—C38—C37	119.0 (3)
C4—C5—C6	119.7 (3)	C39—C38—H38	120.5
C6—C5—H5	120.1	C38—C39—H39	120.0
C1—C6—C7	113.9 (3)	C38—C39—C40	120.0 (3)
C5—C6—C1	120.4 (3)	C40—C39—H39	120.0
C5—C6—C7	125.5 (3)	N4—C40—C39	121.9 (3)
N1—C7—C6	112.9 (3)	N4—C40—H40	119.0
N1—C7—C14	119.5 (3)	C39—C40—H40	119.1
C14—C7—C6	127.6 (3)	N4—C41—C37	121.5 (3)
N1—C8—H8	119.1	N4—C41—C42	116.7 (3)
N1—C8—C9	121.8 (3)	C37—C41—C42	121.8 (3)
C9—C8—H8	119.1	N3—C42—C34	122.0 (3)
C8—C9—H9	120.4	N3—C42—C41	116.8 (2)
C8—C9—C15	119.2 (3)	C34—C42—C41	121.1 (3)
C15—C9—H9	120.4	N5—C43—N6	111.6 (3)
C11—C10—H10	119.5	N5—C43—C44	124.1 (3)
C11—C10—C15	121.1 (3)	N6—C43—C44	124.3 (3)
C15—C10—H10	119.5	C45—C44—C43	118.7 (3)
C10—C11—H11	120.2	C49—C44—C43	122.3 (3)
C10—C11—C12	119.6 (3)	C49—C44—C45	119.0 (3)
C12—C11—H11	120.2	C44—C45—H45	119.9
C11—C12—H12	119.5	C46—C45—C44	120.2 (3)
C13—C12—C11	121.0 (3)	C46—C45—H45	119.9
C13—C12—H12	119.5	C45—C46—H46	119.7
C12—C13—H13	119.6	C47—C46—C45	120.6 (3)
C12—C13—C14	120.8 (3)	C47—C46—H46	119.7
C14—C13—H13	119.6	C46—C47—H47	120.5
C7—C14—C15	118.2 (3)	C46—C47—C48	119.1 (3)
C13—C14—C7	123.9 (3)	C48—C47—H47	120.5
C13—C14—C15	117.8 (3)	C47—C48—H48	119.6
C9—C15—C10	121.5 (3)	C49—C48—C47	120.8 (3)
C9—C15—C14	119.0 (3)	C49—C48—H48	119.6
C10—C15—C14	119.5 (3)	C44—C49—H49	119.8
C17—C16—Ir1	127.0 (2)	C48—C49—C44	120.4 (3)
C17—C16—C21	118.4 (3)	C48—C49—H49	119.8
C21—C16—Ir1	114.5 (2)	F1—P1—F2	87.82 (13)
C16—C17—H17	119.4	F3—P1—F1	178.04 (15)
C18—C17—C16	121.2 (3)	F3—P1—F2	90.34 (13)
C18—C17—H17	119.4	F3—P1—F4	91.62 (13)
C17—C18—H18	119.9	F3—P1—F5	90.58 (14)
C19—C18—C17	120.2 (3)	F3—P1—F6	90.54 (14)
C19—C18—H18	119.9	F4—P1—F1	90.22 (14)
C18—C19—H19	119.9	F4—P1—F2	178.01 (14)
C18—C19—C20	120.1 (3)	F4—P1—F5	90.55 (12)
C20—C19—H19	119.9	F4—P1—F6	90.48 (12)
C19—C20—H20	120.1	F5—P1—F1	90.08 (14)
C19—C20—C21	119.7 (3)	F5—P1—F2	89.12 (12)
C21—C20—H20	120.1	F5—P1—F6	178.46 (14)
C16—C21—C22	114.8 (3)	F6—P1—F1	88.77 (14)
C20—C21—C16	120.1 (3)	F6—P1—F2	89.82 (12)
C20—C21—C22	124.9 (3)	C50—O1—H1	104 (4)
N2—C22—C21	112.9 (3)	O1—C50—H50A	109.5
N2—C22—C29	119.8 (3)	O1—C50—H50B	109.5
C29—C22—C21	127.3 (3)	O1—C50—H50C	109.5
N2—C23—H23	118.8	H50A—C50—H50B	109.5
C24—C23—N2	122.4 (3)	H50A—C50—H50C	109.5
C24—C23—H23	118.8	H50B—C50—H50C	109.5
			
Ir1—N1—C7—C6	−20.0 (3)	C18—C19—C20—C21	−2.6 (5)
Ir1—N1—C7—C14	158.0 (2)	C19—C20—C21—C16	4.9 (5)
Ir1—N1—C8—C9	−164.0 (2)	C19—C20—C21—C22	179.0 (3)
Ir1—N2—C22—C21	−15.8 (3)	C20—C21—C22—N2	−163.2 (3)
Ir1—N2—C22—C29	164.1 (2)	C20—C21—C22—C29	17.0 (5)
Ir1—N2—C23—C24	−172.4 (3)	C21—C16—C17—C18	0.6 (5)
Ir1—N3—C31—C32	175.0 (2)	C21—C22—C29—C28	14.6 (5)
Ir1—N3—C42—C34	−176.5 (2)	C21—C22—C29—C30	−168.3 (3)
Ir1—N3—C42—C41	3.5 (3)	C22—N2—C23—C24	−1.2 (5)
Ir1—N4—C40—C39	177.3 (3)	C22—C29—C30—C24	−7.4 (5)
Ir1—N4—C41—C37	−179.5 (2)	C22—C29—C30—C25	174.3 (3)
Ir1—N4—C41—C42	1.6 (3)	C23—N2—C22—C21	172.4 (3)
Ir1—C1—C2—C3	−172.2 (2)	C23—N2—C22—C29	−7.8 (4)
Ir1—C1—C6—C5	171.1 (2)	C23—C24—C30—C25	177.1 (3)
Ir1—C1—C6—C7	−3.7 (3)	C23—C24—C30—C29	−1.1 (5)
Ir1—C16—C17—C18	−176.0 (2)	C25—C26—C27—C28	−5.8 (6)
Ir1—C16—C21—C20	173.2 (2)	C26—C25—C30—C24	−174.4 (4)
Ir1—C16—C21—C22	−1.5 (3)	C26—C25—C30—C29	3.8 (5)
N1—C7—C14—C13	−166.2 (3)	C26—C27—C28—C29	0.9 (5)
N1—C7—C14—C15	9.2 (4)	C27—C28—C29—C22	−176.8 (3)
N1—C8—C9—C15	4.3 (5)	C27—C28—C29—C30	6.1 (5)
N2—C22—C29—C28	−165.2 (3)	C28—C29—C30—C24	169.9 (3)
N2—C22—C29—C30	12.0 (4)	C28—C29—C30—C25	−8.4 (5)
N2—C23—C24—C30	5.7 (5)	C30—C25—C26—C27	3.4 (6)
N3—C31—C32—C33	1.2 (5)	C31—N3—C42—C34	−0.4 (4)
N4—C41—C42—N3	−3.4 (4)	C31—N3—C42—C41	179.5 (3)
N4—C41—C42—C34	176.5 (3)	C31—C32—C33—C34	−0.8 (5)
N5—C35—C36—N6	0.3 (3)	C32—C33—C34—C35	−179.4 (3)
N5—C35—C36—C37	179.0 (3)	C32—C33—C34—C42	−0.2 (4)
N5—C43—C44—C45	11.6 (5)	C33—C34—C35—N5	1.1 (5)
N5—C43—C44—C49	−167.0 (3)	C33—C34—C35—C36	−179.5 (3)
N6—C36—C37—C38	−2.0 (6)	C33—C34—C42—N3	0.8 (4)
N6—C36—C37—C41	176.7 (3)	C33—C34—C42—C41	−179.2 (3)
N6—C43—C44—C45	−169.9 (3)	C34—C35—C36—N6	−179.2 (3)
N6—C43—C44—C49	11.4 (5)	C34—C35—C36—C37	−0.5 (5)
C1—C2—C3—C4	0.5 (5)	C35—N5—C43—N6	0.8 (3)
C1—C6—C7—N1	15.4 (4)	C35—N5—C43—C44	179.5 (3)
C1—C6—C7—C14	−162.3 (3)	C35—C34—C42—N3	−179.9 (3)
C2—C1—C6—C5	−3.3 (4)	C35—C34—C42—C41	0.2 (4)
C2—C1—C6—C7	−178.0 (3)	C35—C36—C37—C38	179.7 (3)
C2—C3—C4—C5	−0.7 (5)	C35—C36—C37—C41	−1.6 (4)
C3—C4—C5—C6	−1.2 (5)	C36—N6—C43—N5	−0.7 (4)
C4—C5—C6—C1	3.1 (5)	C36—N6—C43—C44	−179.3 (3)
C4—C5—C6—C7	177.3 (3)	C36—C37—C38—C39	175.9 (3)
C5—C6—C7—N1	−159.0 (3)	C36—C37—C41—N4	−175.9 (3)
C5—C6—C7—C14	23.2 (5)	C36—C37—C41—C42	3.0 (4)
C6—C1—C2—C3	1.5 (4)	C37—C38—C39—C40	0.9 (5)
C6—C7—C14—C13	11.4 (5)	C37—C41—C42—N3	177.7 (3)
C6—C7—C14—C15	−173.2 (3)	C37—C41—C42—C34	−2.3 (5)
C7—N1—C8—C9	4.7 (5)	C38—C37—C41—N4	3.0 (5)
C7—C14—C15—C9	−0.6 (4)	C38—C37—C41—C42	−178.2 (3)
C7—C14—C15—C10	−179.6 (3)	C38—C39—C40—N4	1.1 (5)
C8—N1—C7—C6	170.6 (3)	C40—N4—C41—C37	−1.1 (5)
C8—N1—C7—C14	−11.4 (4)	C40—N4—C41—C42	−179.9 (3)
C8—C9—C15—C10	173.0 (3)	C41—N4—C40—C39	−1.0 (5)
C8—C9—C15—C14	−6.0 (4)	C41—C37—C38—C39	−2.8 (5)
C10—C11—C12—C13	−3.7 (5)	C42—N3—C31—C32	−0.6 (4)
C11—C10—C15—C9	−175.6 (3)	C42—C34—C35—N5	−178.1 (3)
C11—C10—C15—C14	3.4 (5)	C42—C34—C35—C36	1.2 (4)
C11—C12—C13—C14	3.1 (5)	C43—N5—C35—C34	178.7 (3)
C12—C13—C14—C7	176.1 (3)	C43—N5—C35—C36	−0.7 (3)
C12—C13—C14—C15	0.7 (5)	C43—N6—C36—C35	0.3 (3)
C13—C14—C15—C9	175.1 (3)	C43—N6—C36—C37	−178.3 (3)
C13—C14—C15—C10	−3.9 (4)	C43—C44—C45—C46	−176.4 (3)
C15—C10—C11—C12	0.4 (5)	C43—C44—C49—C48	177.0 (3)
C16—C17—C18—C19	1.7 (5)	C44—C45—C46—C47	−1.4 (5)
C16—C21—C22—N2	11.2 (4)	C45—C44—C49—C48	−1.7 (5)
C16—C21—C22—C29	−168.6 (3)	C45—C46—C47—C48	−0.1 (6)
C17—C16—C21—C20	−3.9 (5)	C46—C47—C48—C49	0.7 (6)
C17—C16—C21—C22	−178.6 (3)	C47—C48—C49—C44	0.2 (6)
C17—C18—C19—C20	−0.7 (5)	C49—C44—C45—C46	2.3 (5)

### Bis[2-(isoquinolin-1-yl)phenyl-κ2N,C1](2-phenyl-1H-imidazo[4,5-f][1,10]phenanthroline-κ2N,N')iridium(III) hexafluoridophosphate methanol monosolvate Hydrogen-bond geometry (Å, º)

**Table:**

*D*—H···*A*	*D*—H	H···*A*	*D*···*A*	*D*—H···*A*
N6—H6···F1	0.85 (5)	2.28 (5)	3.110 (4)	166 (4)
N6—H6···F2	0.85 (5)	2.57 (4)	3.249 (4)	139 (4)
O1—H1···N5	0.93 (7)	1.92 (7)	2.849 (4)	175 (6)

## References

[bb1] Dolomanov, O. V., Bourhis, L. J., Gildea, R. J., Howard, J. A. K. & Puschmann, H. (2009). *J. Appl. Cryst.* **42**, 339–341.

[bb2] Kang, T.-S., Mao, Z., Ng, C.-T., Wang, M., Wang, W., Wang, C., Lee, S. M.-Y., Wang, Y., Leung, C.-H. & Ma, D.-L. (2016). *J. Med. Chem.* **59**, 4026–4031. 10.1021/acs.jmedchem.6b00112 27054262

[bb3] Ma, Y., Xu, H., Zeng, Y., Ho, C.-L., Chui, C.-H., Zhao, Q., Huang, W. & Wong, W.-Y. (2015). *J. Mater. Chem. C.* **3**, 66–72.

[bb4] Rigaku OD (2023). *CrysAlis PRO*. Rigaku Corporation, Yarnton, England.

[bb5] Sheldrick, G. M. (2015*a*). *Acta Cryst.* A**71**, 3–8.

[bb6] Sheldrick, G. M. (2015*b*). *Acta Cryst.* C**71**, 3–8.

[bb7] Zhao, Q., Liu, S., Shi, M., Li, F., Jing, H., Yi, T. & Huang, C. (2007). *Organometallics*, **26**, 5922–5930.

[bb8] Zhao, Q., Liu, S., Shi, M., Wang, C., Yu, M., Li, L., Li, F., Yi, T. & Huang, C. (2006). *Inorg. Chem.* **45**, 6152–6160. 10.1021/ic052034j 16878924

